# Human adipose tissue expansion in pregnancy is impaired in gestational diabetes mellitus

**DOI:** 10.1007/s00125-015-3662-0

**Published:** 2015-06-13

**Authors:** Raziel Rojas-Rodriguez, Lawrence M. Lifshitz, Karl D. Bellve, So Yun Min, Jacqueline Pires, Katherine Leung, Crina Boeras, Aylin Sert, Jacqueline T. Draper, Silvia Corvera, Tiffany A. Moore Simas

**Affiliations:** Program in Molecular Medicine, University of Massachusetts Medical School, 373 Plantation Street, Worcester, MA 01605 USA; Graduate School of Biomedical Sciences, University of Massachusetts Medical School, Worcester, MA USA; Biomedical Imaging Group, University of Massachusetts Medical School, Worcester, MA USA; School of Medicine, University of Massachusetts Medical School, Worcester, MA USA; Clinical Translational Research Pathway, University of Massachusetts Medical School, Worcester, MA USA; Department of Obstetrics & Gynecology, Division of Research, University of Massachusetts Medical School/UMass Memorial Health Care, Memorial Campus - 119 Belmont Street, Worcester, MA 01605 USA; Department of Pediatrics, University of Massachusetts Medical School/UMass Memorial Health Care, Worcester, MA USA

**Keywords:** Adiposity, Endothelial cell, FIJI, IGF-1, IGF-2, Insulin resistance, Vascularisation, Visceral fat

## Abstract

**Aims/hypothesis:**

During pregnancy, adipose tissue (AT) must expand to support the growing fetus and the future nutritional needs of the offspring. Limited expandability of AT is associated with insulin resistance, attributed to ectopic lipid deposition. This study aimed to investigate human AT expandability during pregnancy and its role in the pathogenesis of gestational diabetes mellitus (GDM).

**Methods:**

This cross-sectional study of omental (OM) and subcutaneous (SQ) AT collected at Caesarean delivery included 11 pregnant and three non-pregnant women with normal glucose tolerance (NGT), five with GDM, three with type 2 diabetes mellitus. Adipocyte size, capillary density, collagen content and capillary growth were measured. Affymetrix arrays and real-time PCR studies of gene expression were performed.

**Results:**

Mean OM adipocyte size was greater in women with GDM than in those with NGT (*p* = 0.004). Mean OM and SQ capillary density was lower in GDM compared with NGT (*p* = 0.015). Capillary growth did not differ significantly between groups. The most differentially expressed AT transcript when comparing non-pregnant and pregnant women corresponded to the IGF binding protein (IGFBP)-5, the expression levels of which was found by subsequent quantitative real-time PCR to be lower in women with GDM vs women with NGT (*p* < 0.0001).

**Conclusions/interpretation:**

The relative OM adipocyte hypertrophy and decreased OM and SQ capillary density are consistent with impaired AT expandability in GDM. The induction of adipose tissue IGFBP5 in pregnancy and its decrease in GDM point to the importance of the IGF-1 signalling pathway in AT expansion in pregnancy and GDM susceptibility.

**Electronic supplementary material:**

The online version of this article (doi:10.1007/s00125-015-3662-0) contains peer-reviewed but unedited supplementary material, which is available to authorised users.

## Introduction

Gestational diabetes mellitus (GDM) affects 240,000 pregnancies in the USA each year, a number expected to increase to ~720,000 with changes in diagnostic criteria and increased incidence [1–3]. GDM has been associated with intergenerational effects on risk of obesity, the metabolic syndrome and cardio-metabolic disorders [4–7]. Of women with a history of GDM, 50% develop type 2 diabetes within 5 years [8], and up to one-third of women with type 2 diabetes have a history of GDM [9]. Notwithstanding the considerable public health relevance of this disorder, its aetiology remains unclear.

Appropriate expandability of adipose tissue (AT) is necessary for preventing ectopic deposition of lipids and their metabolites in tissues such as muscle, liver and pancreas, with consequent development of insulin resistance [10–13]. The need for expandability is seen in mouse models in which increased AT mass results in metabolic improvement [14], and in epidemiological studies linking increased subcutaneous (SQ) AT with protection from type 2 diabetes risk [15]. Notably, while SQ AT expansion is associated with diminished risk, expansion of visceral AT is associated with increased risk of metabolic disease [15]. This dichotomy is attributed to the propensity of visceral AT to undergo inflammation [16], associated with insufficient SQ AT expandability [17]. During pregnancy, AT must rapidly expand to support the needs of the fetus and the future needs of the offspring through lactation [18, 19]. Thus, inherent limitations in the ability of AT to expand may be unmasked during pregnancy and manifest as GDM and future susceptibility to type 2 diabetes.

It was thought that GDM arises from placental hormones that induce insulin resistance, coupled with insufficient insulin secretion. Numerous placental hormones can affect insulin sensitivity [20]. However, an association between these and insulin sensitivity in late pregnancy, and with GDM, has not been consistently discerned [21]. A major factor produced by placenta is pregnancy-associated plasma protein-A (PAPP-A), a protease that can hydrolyse IGF binding proteins (IGFBPs) 4 and 5 [22]. IGFBPs bind IGF-1 and/or IGF-2, and control their bioavailability within tissues [23]. The high affinity of IGFBPs for IGFs precludes their binding to the IGF-1 receptor [23, 24]. Thus, through the production of PAPP-A, the placenta can exert a major effect on the growth of tissues through hydrolysis of IGFBPs and release of bound IGF-1. Obesity can affect the placenta, resulting in structural and biochemical alterations [25–28], with potential consequent maternal AT growth.

To explore the role of AT expandability in the aetiology of GDM we conducted a cross-sectional analysis of AT from pregnant women undergoing elective Caesarean deliveries. Our results are consistent with the hypothesis that AT expandability is decreased in individuals with GDM. Moreover, global expression analyses reveal that IGFBP5 is highly induced in AT during pregnancy and is decreased in GDM, suggesting a mechanism for placenta–AT communication that may be impaired in GDM.

## Methods

### Study setting and participants

All pregnant women with singleton gestations presenting to UMass Memorial Health Care (UMMHC) between March 2013 and June 2014 for scheduled Caesarean delivery were considered for enrolment. The Institutional Review Board of the University of Massachusetts Medical School (UMMS) approved the study, and all participants provided informed consent. Exclusion criteria were as follows: (1) pre-gestational type 1 diabetes; (2) underweight pre-pregnancy BMI (<18.5 kg/m^2^); (3) multiple gestations; (4) reported use of substances, including alcohol and/or illicit drugs and replacement or maintenance products, in pregnancy; (5) HIV; (6) hepatitis; (7) age <18 years; (8) autoimmune disease and/or chronic steroid use; (9) prenatal care initiation after 13 completed weeks gestational age and (10) plans to move out of the area within the study period.

### Diabetes status

Type 2 diabetes was diagnosed based on patient report of medical history and confirmatory review of medical records. Individuals were considered to have GDM if they did not have pre-gestational type 1 or type 2 diabetes and either met American Diabetes Association criteria (a value of ≥7.7 mmol/L (140 mg/dl) 1 h after a 50 g dextrose load (Thermoscientific, Middletown, VA, USA) screening test followed by and two or more abnormal values for a 100 g 3 h glucose tolerance test) at routine screening between 24 and 28 weeks gestation, or had a value >10 mmol/l (180 mg/dl) on the 1 h screening test [29, 30].

### Covariates and other relevant data collection

Clinical and anthropometric data were collected at enrolment. Pre-pregnancy BMI was calculated as pre-pregnancy weight (kg)/height^2^ (m^2^). Post-pregnancy BMI was calculated from the last prenatal weight record minus the weight of the baby. Blood glucose was measured 1 h after ingestion of a 50 g glucose load, a test routinely performed for GDM screening in individuals without pre-gestational type 2 diabetes.

### Non-pregnant individuals

Cohorts for Affymetrix arrays were from non-diabetic individuals undergoing bariatric surgery, as previously described [31]. Consent for collection of additional samples of AT was obtained from non-pregnant individuals undergoing panniculectomy surgery who had no history of type 2 diabetes.

### AT analysis

Biological specimens were collected at the time of surgical delivery. After delivery of the baby, two segments (1 cm × 1 cm) of omental (OM) tissue from the midline inferior periphery were collected. After the rectus fascia was re-approximated, prior to skin closure, two samples (1 cm × 1 cm) of SQ AT were obtained from within the surgical incision usually placed approximately 2 cm above the pubic bone (Pfannenstiel incision). In the case of repeat Caesarean deliveries, subcutaneous AT biopsies were taken from deep within the incision to decrease scar tissue sampling.

### Analysis of adipocyte size and collagen staining

**Fig. 1:**
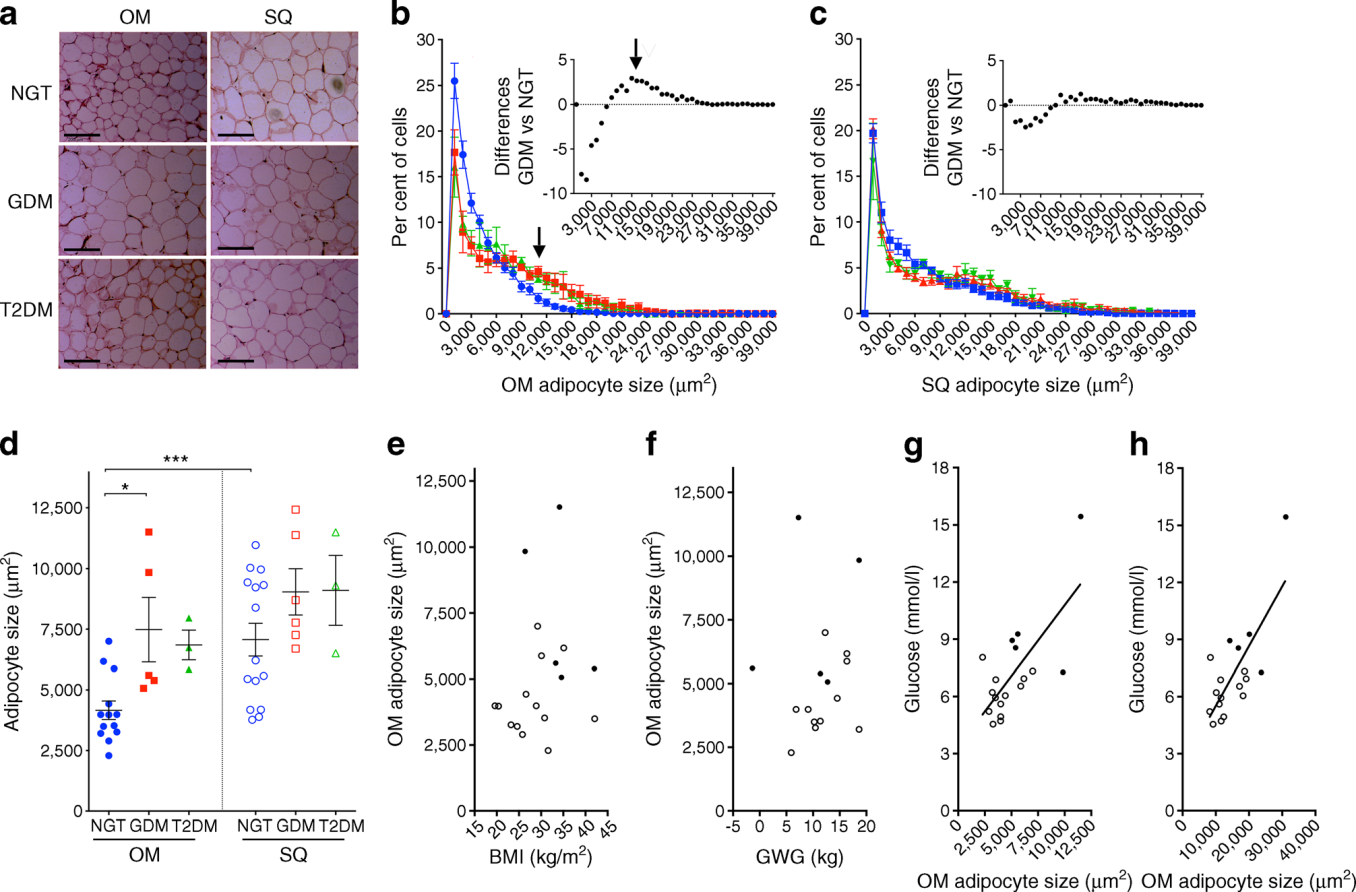
Analysis of adipocyte size in OM and abdominal SQ AT. (**a**) Representative images of H&E-stained AT taken from individuals with NGT, GDM or type 2 diabetes (T2DM). Scale bar, 200 μm. (**b**, **c**). Size distribution of adipocytes from OM (**b**) or SQ (**c**) AT. Plots show the means and SEM at each bin size of women with NGT (blue circles), GDM (red squares) and type 2 diabetes (green triangles). Arrow points to peak in region of larger adipocyte size in tissues from GDM and type 2 diabetes. Insets depict the paired signed rank difference between the histograms. (**d**) Mean adipocyte size from OM (coloured symbols) and SQ (white symbols) AT from women with NGT (blue circles), GDM (red squares) and type 2 diabetes (green triangles). Symbols show the means of each individual and lines represent the means and SEM of all individuals. *p < 0.05 and ***p < 0.001 for indicated comparisons. (**e**–**h**) Linear regression analyses: BMI vs mean OM adipocyte size (*r* ^2^ = 0.038; *p* = 0.433) (**e**); gestational weight gain (GWG) vs mean OM adipocyte size (*r* ^2^ = 0.149; *p* = 0.651) (**f**); mean OM adipocyte size vs serum glucose (*r* ^2^ = 0.505; *p* = 0.0009) (**g**) and maximal OM adipocyte size vs serum glucose (*r* ^2^ = 0.568; *p* = 0.0003) (**h**). White circles, NGT; black circles, GDM

AT samples were fixed in 4% formaldehyde and embedded in paraffin. Tissue sections (8 μm) were mounted on Superfrost Plus microscope slides (Fisher Scientific, Pittsburgh, PA, USA), and stained with haematoxylin and eosin (H&E). Investigators acquiring the images were blinded to the origin of the sample. Adipocyte size was determined using an automated procedure based on the open software platform FIJI [32] as described in electronic supplementary material (ESM) Fig. 1. Collagen was detected using picrosirius red staining. For quantification, images were imported into Image J (http://fiji.sc/Fiji), and converted into 8 bit greyscale images. After background subtraction, total intensity was recorded.

### Analysis of capillary density

Fragments of AT were fixed in 4% formaldehyde, washed and stained with Rhodamine-Lectin UEA-1 (Vector Labs, Burlingame, CA, USA) for 1 h at room temperature and then mounted between 1.5 mm coverslips sealed with Pro-Long Gold Antifade Reagent (Life Technologies, Grand Island, NY, USA). A Zeiss (Peabody, MA, USA) Axiovert 100 inverted microscope with a ×10 objective and AxioCam HRm camera was used to acquire nine Z-plane image stacks at 10 μm intervals. The individual collecting the images was unaware of the group they corresponded to. Quantification of capillary density was determined as described in ESM Fig. 2.

### AT capillary growth assay

AT biopsies were cut into small (1 mm^3^) pieces and embedded in individual wells of a 96-multiwell plate as described [33]. After 11 days of culture in EBM-2 medium supplemented with EGM-2 MV (BulletKit CC-3202; Lonza, Allendale, NJ, USA), images were acquired using a Zeiss Axio Observer Z1 equipped with an automated stage and a Clara High Resolution CCD Camera (Andor, Concord, MA, USA). Images were taken under ×2.5 magnification as a stack composed of five Z planes at 150 μm intervals and as a canvas of four quadrants per well and then combined into a single three-dimensional image. Subsequent image processing and quantification of capillary growth were determined as described in ESM Fig. 3.

### Affymetrix arrays

Total RNA was isolated using TRIzol (Life Technologies). Affymetrix (Santa Clara, CA, USA) protocols were followed for the preparation of cRNA, which was hybridised to HG-U133v2 Chips. Raw expression data collected from an Affymetrix HP GeneArrayScanner was normalised across all data sets using the RMA algorithm. Probes in which any condition resulted in raw expression values above 1,000 were included in the heat map shown in Fig. 3.

### RNA isolation and quantitative real-time PCR

Total RNA was extracted from AT using the RNeasy Lipid Tissue Mini Kit (Qiagen, Valencia, CA, USA). One microgram of total RNA was reverse-transcribed using iScript cDNA synthesis kit (Bio-Rad, Hercules, CA, USA). cDNA was used as template for real-time PCR using the iQ SYBR Green Supermix kit (Bio-Rad) and the CFX96 Real-Time System (Bio-Rad). Human ribosomal protein L4 (RPL4) was used for normalisation. To reflect the abundance of the different IGFBPs and IGF-1 relative to each other and between groups, all values were normalised to the content of the lowest expressed transcript, which was IGFBP1 in the non-pregnant group.

### Statistical analysis

Clinical study data were collected and managed using REDCap (Research Electronic Data Capture). Stata/MP v. 13.1 (http://www.stata.com/statamp) or GraphPad Prism v. 6.0 (www.graphpad.com/scientific-software/prism) were used for analysis as indicated. Multiple measures for each individual were acquired for each variable investigated. For adipocyte size data, between five and ten sections per each individual were used to acquire between 38 and 117 images. For capillary density data three three-dimensional image stacks were acquired from three independent sections per each individual. For capillary growth data, between seven and 58 observations were made for each individual. For picrosirius red staining, three sections per individual were used to acquire 15 images. Each individual’s data were collapsed to the mean, which was then used for group analysis. A sensitivity analysis was performed with both the mean and median value for each individual. Sensitivity analysis results were similar to the mean, therefore we present only the mean results. To calculate the maximum adipocyte size, the single largest adipocyte in each field was recorded, and the mean value calculated for each individual. Categorical variables were described using frequency and percentage with Fisher’s exact test used to compare groups. Group comparisons for adipocyte size, capillary density and angiogenesis data were made using the Wilcoxon rank sum test (Mann–Whitney test in Prism 6.0). Within-subject differences between OM and SQ tissue were determined using the Wilcoxon signed-rank test. Histograms of adipocyte size distributions for each patient were generated using the same bin sizes, and groups of histograms were compared using the Wilcoxon test for histograms (Prism 6.0). Statistical analysis of difference between groups in experiments involving real-time PCR was done using two-way ANOVA with the Holms–Sidak correction for multiple comparisons.

## Results

### Patient characteristics

Relevant characteristics of the patients used for adipocyte size, capillary density, fibrosis and capillary growth assays are summarised in Table 1. Because the number of individuals in the type 2 diabetes category was small, comparisons were made only between the NGT and GDM groups. A trend for higher pre-pregnancy and post-delivery BMI in individuals with GDM was noted but the difference was not statistically significant. While there was a statistically significant difference in age between the groups, none of the observed variables correlated with this variable. Nevertheless, the small sample size is a limitation of this study.

**Table 1:** Characteristics of study participants

Characteristic	NGT (*n* = 15)	GDM (*n* = 6)	Type 2 diabetes (*n* = 3)	*p* value (NGT vs GDM)
BMI pre-pregnancy (kg/m^2^)	27.3 (6.2)	32.2 (6.2)	34.0 (6.6)	0.0895
BMI at term (kg/m^2^)	30.6 (5.8)	33.8 (5.3)	37.4 (5.9)	0.241
Age (years)	29.7 (4.1)	35.3 (4.6)	32.3 (5.5)	0.0119
GWG (kg)	12.65 (3.9)	9.4 (6.1)	11.0 (4.4)	0.1531
GWG–baby (kg)	9.1 (3.8)	5.8 (5.9)	7.9 (4.4)	0.1292
Baby weight (kg)	3.53 (0.47)	3.68 (0.438)	3.07 (0.166)	0.7826
Serum glucose (mmol/l)^a^	7.13 (2.29)	9.36 (2.78)	N/A	0.0003

Data are presented as mean (SD)

^a^ Serum glucose measured 1 h after 50 g dextrose load screening test

GWG, gestational weight gain; GWG–baby, gestational weight gain immediately after delivery of baby

### Adipocyte size, capillary density and angiogenic potential

Adipocyte size was quantified (Fig. 1a). The mean cell size in OM AT was 4,163 μm^2^ (SD 1,380 μm^2^; *n* = 13) in individuals with NGT, 7,482 μm^2^ (SD 2,980 μm^2^; *n* = 5) in those with GDM and 6,849 μm^2^ (SD 1,060 μm^2^; *n* = 3) in those with type 2 diabetes, with a statistically significant difference between the NGT and GDM group (*p* = 0.019). OM AT from women with GDM contained an increased number of large adipocytes (as shown by a peak in the size distribution histogram above the mean value in Fig. 1b, arrows). The difference in the frequency distribution between NGT and GDM was statistically significant (*p* = 0.028). A similar trend was seen in samples from three type 2 diabetes cases, also plotted but not analysed due to the small sample size. The mean cell size in SQ AT was 7,066 μm^2^ (SD 2,612 μm^2^; *n* = 15) in individuals with NGT, 9,045 μm^2^ (SD 2,340 μm^2^; *n* = 5) in those with GDM and 9,102 μm^2^ (SD 2,503 μm^2^; *n* = 3) in those with type 2 diabetes. The difference between the NGT and GDM group was not statistically significant (*p* = 0.178). A trend for larger adipocyte numbers in SQ tissue from individuals with GDM was seen in the size distribution histograms (Fig. 1c), but the difference did not reach statistical significance. In women with NGT, adipocytes from OM AT (mean size 4,178 μm^2^; SD 1,440 μm^2^; *n* = 12) were significantly smaller (*p* = 0.0005) than those from SQ AT (mean size 7,581 μm^2^; SD 2,672 μm^2^; *n* = 12); this difference was eliminated in GDM (*p* = 0.125) due to the increase in mean OM adipocyte size (mean size 7,482 μm^2^; SD 2,980 μm^2^; *n* = 5) (Fig. 1d). The increase in mean OM adipocyte size in individuals with GDM could not be explained by an increase in BMI (Fig. 1e) or gestational weight gain (Fig. 1f) but was highly correlated with serum glucose levels (Fig. 1g), as was the maximal OM adipocyte size (Fig. 1h). This finding is consistent with those of previous studies in non-pregnant individuals in which large adipocytes are associated with dyslipidaemia as well as glucose and insulin abnormalities [34, 35], revealing a similarity between a central AT feature in GDM and type 2 diabetes.

**Fig. 2:**
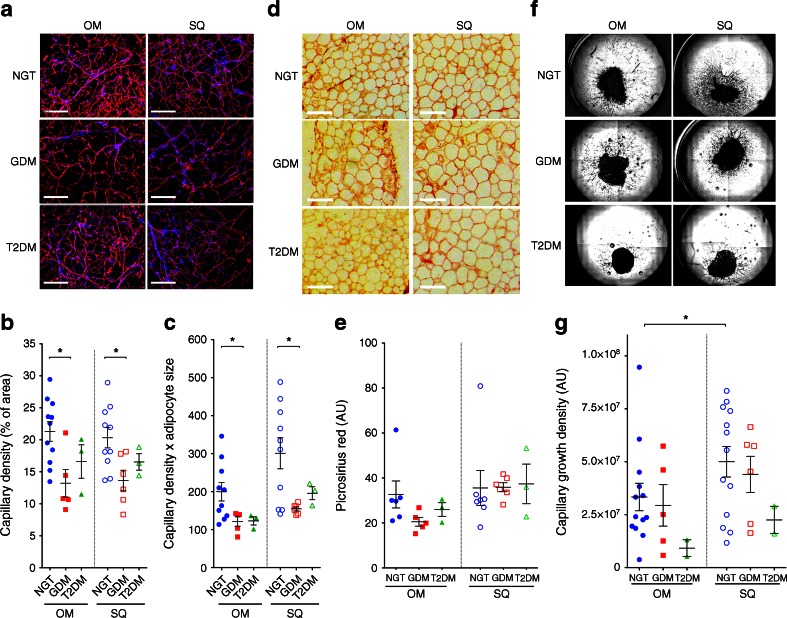
Analysis of capillary density, collagen content and angiogenic potential in OM and abdominal SQ AT. (**a**) Representative images of capillary density in Rhodamine-lectin UEA-1-stained AT taken from individuals with NGT, GDM or type 2 diabetes (T2DM). Scale bar, 200 μm. (**b**, **c**) Capillary density, shown as % of area (**b**) and as % of area × mean adipocyte size (**c**), in OM (coloured symbols) and SQ (white symbols) AT taken from individuals with NGT (circles), GDM (squares) and type 2 diabetes (triangles). Symbols show the means for each individual and lines represent the mean and SEM of all individuals. *p < 0.05 for indicated comparisons. (**d**) Representative examples of AT sections stained with picrosirius red. (**e**) Intensity of picrosirius red staining; symbols and lines are as described in (**b**) and (**c**). (**f**) Representative examples of capillary growth in AT. (**g**) Capillary growth area density; symbols and lines are as described in (**b**) and (**c**). *p < 0.05 for indicated comparisons

In parallel with the growth of adipocytes, the capillary network of AT must expand to sustain adipocyte function [36, 37]. Moreover, the vasculature of AT is a developmental niche for adipocyte precursors [38] and adequate angiogenesis may be a prerequisite for adipocyte hyperplasia. Capillary density was measured in lectin-stained whole mounts (Fig. 2a–c). The mean area of lectin staining (as % of total area) in OM AT was 21.3% (SD 5.0%; *n* = 11) in individuals with NGT, 13.2% (SD 4.8%; *n* = 5) in those with GDM and 16.6% (SD 4.5%; *n* = 3) in those with type 2 diabetes, with a statistically significance difference between the NGT and GDM group (*p* = 0.013). The mean area of lectin staining in SQ AT was 20.3% (SD 5.0%; *n* = 18) in individuals with NGTs, 13.6% (SD 3.9%; *n* = 5) in those with GDM and 16.5% (SD 2.2%; *n* = 3) in those with type 2 diabetes, with a statistically significant difference between the NGT and GDM group (*p* = 0.039). Differences in capillary density could not be attributed to changes in adipocyte size, as the product of capillary density and adipocyte size remained significantly different (Fig. 2c). To explore other alterations in AT associated with metabolic disease, such as fibrosis [39], we analysed fixed sectioned tissue for collagen content using picrosirius red (Fig. 2d, e). No significant differences were detected between groups, possibly due to the lower BMI and early onset of metabolic disease in this population compared with previously studied bariatric surgery populations [39, 40].

To gain insight into the causes that may underlie the observed changes in capillary density seen in GDM we measured the capacity of AT to form capillaries ex vivo (Fig. 2f). This assay measures the competence of endothelial cells and cells associated with the vasculature to grow under ideal angiogenic conditions where growth factors are optimally supplied in the growth medium. Thus, it can help distinguish between the effects of environmental factors (e.g. decreased levels of growth factors) and those of other inherent properties of the tissue (e.g. decreased number of progenitor cells). The mean growth density (number of pixels) for SQ and OM AT from individuals with NGT was 5.32 × 10^7^ (SD 2.43 × 10^7^; *n* = 12) and 3.34 × 10^7^ (SD 2.34 × 10^7^; *n* = 13), respectively, with a statistically significant difference (*p* = 0.033), consistent with our previous observations in non-pregnant obese individuals [31]. However, no significant difference (*p* = 0.882) was detected in OM AT when comparing individuals with NGT (3.34 × 10^7^; SD 2.34 × 10^7^; *n* = 13) and those with GDM (2.94 × 10^7^; SD 2.19 × 10^7^; *n* = 5) (Fig. 2g). Also, no significant difference (*p* = 0.349) was detected when comparing SQ AT explants from individuals with NGT (5.32 × 10^7^; SD 2.43 × 10^7^; *n* = 12) with those from individuals with GDM (4.40 × 10^7^; SD 2.09 × 10^7^; *n* = 6). The lower mean angiogenic potential seen in tissues from two individuals with type 2 diabetes must be confirmed with a larger sample size.

### Gene expression analysis

**Fig. 3:**
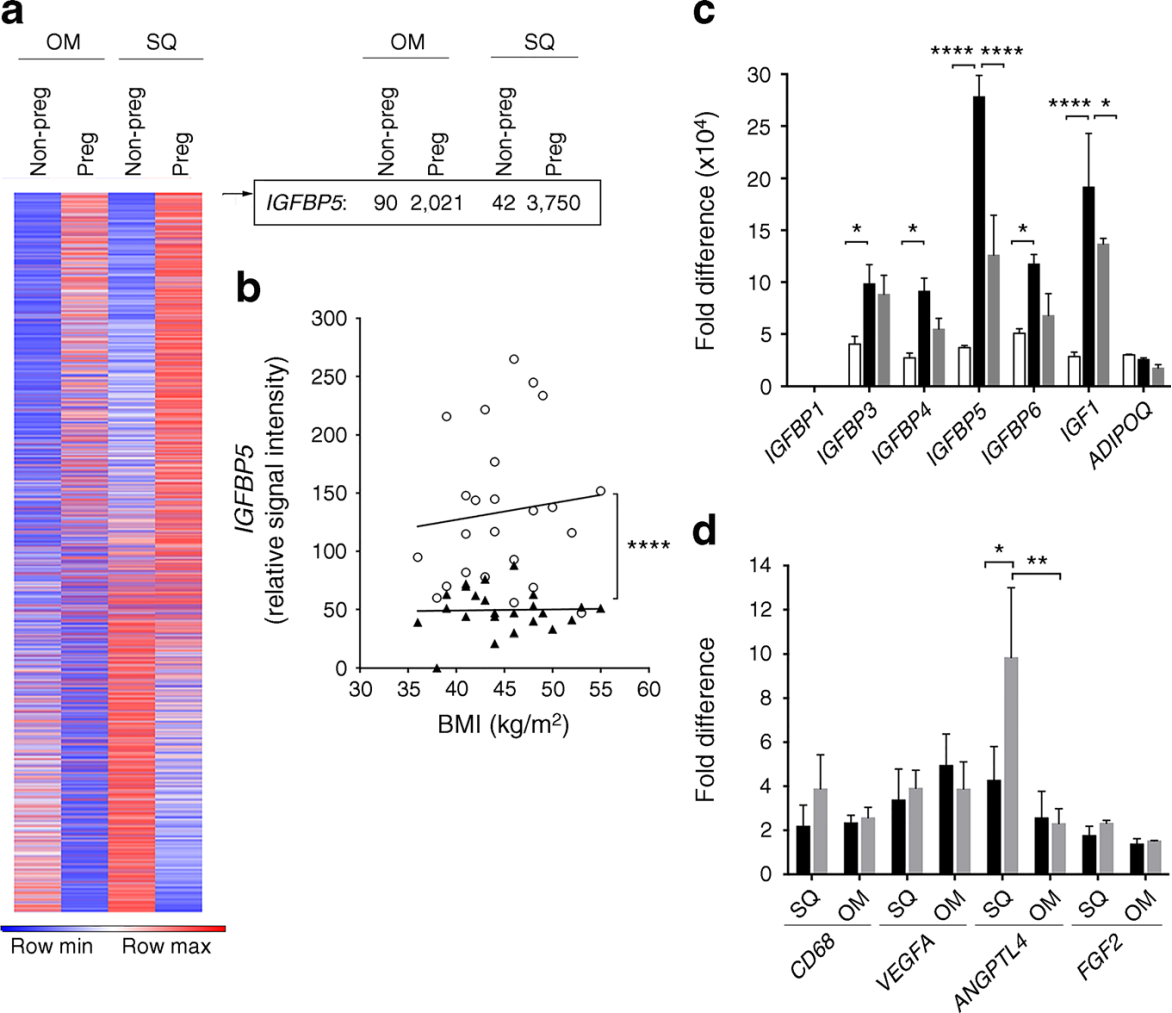
AT gene expression in pregnancy and GDM. (**a**) Heat map of 22,000 genes, and specific expression values for *IGFBP5*, in AT from non-pregnant and pregnant women with NGT. Values for *IGFBP5* (relative signal intensity) are shown. (**b**) Relationship between BMI and *IGFBP5* levels in SQ (black triangles) and OM (white circles) AT from individuals used for the Affymetrix array. (c) Real-time PCR analysis of SQ AT from non-pregnant women with NGT (white bars), pregnant women with NGT (black bars) and pregnant women with GDM (grey bars). Values are normalised to the lowest expressed transcript in the NGT group, which was *IGFBP1*. (**d**) Real-time PCR analysis of SQ AT from pregnant women with NGT (black bars) and pregnant women with GDM (grey bars). Values are normalised to the lowest expressed transcript in the NGT group, which was *FGF2*. Adjusted *p* values: *p < 0.05, **p < 0.01 and ****p < 0.0001 for indicated comparisons

To explore the mechanisms involved in AT growth in pregnancy, we compared previously acquired [31] Affymetrix gene arrays of SQ and OM AT from 11 non-pregnant, non-diabetic women undergoing bariatric surgery [31], with similar arrays of pregnant women with NGT (*n* = 3 individuals; BMI 20.4, 19.6 and 19.5 kg/m^2^). Numerous differences in gene expression were seen between OM and SQ AT depots, and between pregnant and non-pregnant groups (Fig. 3a). The most differentially expressed gene when comparing AT from non-pregnant and pregnant women, for both OM and SQ AT, was *IGFBP5*. Further analysis of Affymetrix data revealed higher levels of *IGFBP5* in the OM AT than in the SQ AT of non-pregnant women, with no correlation with BMI (Fig. 3b). No changes in expression of *IGFBP5* have been observed in SQ AT arrays from individuals with a BMI range of 16.7–50.2 kg/m^2^ and normal or impaired glucose tolerance [41] (data set record GDS3961), supporting the notion that the induction seen in our study was due to pregnancy. The most significantly altered genes (*p* < 0.0001) were used to identify pathways significantly associated with pregnancy. Kyoto Encyclopaedia of Genes and Genomes (KEGG) identified the ‘protein processing in the endoplasmic reticulum’ and ‘protein export’ pathways as being enriched in differentially expressed genes (ESM Table 1).

To verify the arrays, we conducted real-time PCR analysis of SQ AT from additional cohorts of non-pregnant women undergoing panniculectomy surgery (mean BMI 29.6 kg/m^2^; SEM 1.2 kg/m^2^; *n* = 3), pregnant women with NGT (mean BMI 19.7 kg/m^2^; SEM 0.1 kg/m^2^; *n* = 3) and pregnant women with GDM (mean BMI 34 kg/m^2^; SEM 4.6 kg/m^2^, *n* = 3) (Fig. 3c). These results confirmed the marked induction of *IGFBP5* by pregnancy seen in Affymetrix arrays and revealed a significant decrease in *IGFBP5* and *IGF1* expression in tissue from individuals with GDM. While this difference also coincided with a significant difference in BMI among the groups, the lack of correlation between *IGFBP5* expression and BMI in the individuals used for Affymetrix gene chip analysis (Fig. 3b), and between *IGFBP5* expression and the BMI of non-pregnant individuals and those with GDM (*r*^2^ = 0.28, *p* = 0.278), suggests that the observed differences in *IGFBP5* expression between individuals with NGT and GDM is independent of BMI. In addition to differences in *IGFBP5*, differences in the expression of *IGF1*, *IGFBP3*, *IGFBP4* and *IGFBP6* were seen (Fig. 3c), revealing alterations in the IGF-1 signalling axis in AT in response to pregnancy and GDM. Moreover, the decrease in *IGFBP5* and *IGF1* expression seen in GDM was not paralleled by changes in other key angiogenic factor genes, such as *VEGFA* and *FGF2*, nor by significant inflammatory alterations as inferred by unchanged gene expression of the macrophage marker CD-68 (Fig. 3d).

## Discussion

Adipocyte size is an indicator of the mode of AT expansion, which can occur through two distinct processes: adipocyte hypertrophy, where each adipocyte increases in size to contain a larger lipid droplet, and hyperplasia, where more adipocytes form through differentiation of precursor cells [34, 42]. AT hyperplasia (small adipocytes) is significantly associated with better glucose, insulin and lipid profiles [34, 35]. In contrast, large adipocytes are associated with dyslipidaemia, glucose and insulin abnormalities [34, 35], are insulin resistant [43] and are prone to necrosis and inflammation. Hyperplasia may be more effective at increasing total AT mass and storage capacity, thus preventing deposition of deleterious lipid species in other organs and ensuing insulin resistance. Consistent with this possibility are data from obese mouse models in which AT containing small adipocytes leads to improved glucose homeostasis [14, 44]. The relative hypertrophy of adipocytes in women with GDM, together with their trend to a lower gestational weight gain (Table 1), suggests a decreased capacity for hyperplastic adipocyte growth and AT expansion, with consequent negative effects on glucose and insulin homeostasis.

Adipocyte progenitors are tightly associated with the AT capillary network [45, 46]. Thus, the decreased capillary density seen in individuals with GDM could underlie diminished pre-adipocyte proliferation and hyperplastic growth, with consequent hypertrophy of existing adipocytes. Insufficient vascular growth in the AT of individuals with GDM could also contribute to insulin resistance through hypoxia [31, 47]. Expression levels of the gene encoding angiopoietin-like 4 (ANGPTL-4), which is sensitive to hypoxia [48], were found to be elevated in GDM, possibly indicating insufficient oxygenation. Other mechanisms could include failure of nutrient exchange or failure to deliver secreted hormones (adipokines) into the circulation.

**Fig. 4:**
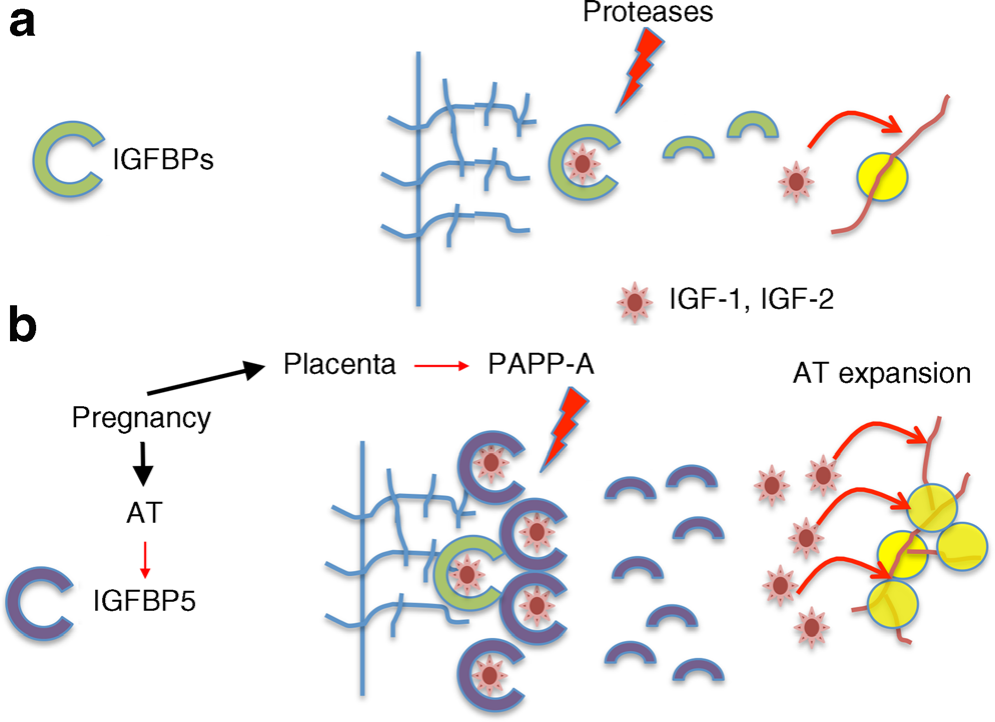
Potential role of IGFBP5 in AT expansion in pregnancy. (**a**) In non-pregnant women, IGFBPs sequester IGF-1 and IGF-2. Proteolysis of IGFBPs releases the growth factors, which act on AT vasculature and maintain tissue homeostasis. (**b**) In pregnancy, the induction of IGFBP5 increases the amount of sequestered IGF-1. The pregnancy-specific protease PAPP-A degrades IGFBP5 to release IGFs, promoting angiogenesis, pre-adipocyte proliferation and hyperplastic expansion

Previous studies from our group identified IGFBP4 as a potential key factor in AT expansion in response to a high-fat diet in mice [49]. The marked upregulation of *IGFBP5* seen in AT from pregnant women supports a similar role for the IGF-1 signalling pathway in adult AT expansion. IGFBP5 binds IGF-1 and IGF-2 with high affinity and has been shown in cell and animal models to inhibit signalling by sequestering these growth factors [23]. However, IGFBP5 also binds to the extracellular matrix of tissues, and can thereby serve as a local reservoir of IGF-1 [23]. The concurrent expression of IGFBP5 in AT and secretion of PAPP-A by the placenta suggests a mechanism whereby the local concentration of IGF-1 is increased in AT through interaction with IGFBP5 and release in response to PAPP-A secretion, thereby coordinating placental function with maternal AT expansion (Fig. 4). Abnormalities in this mechanism could potentially lead to impaired AT expansion and to lipotoxicity and metabolic disruption manifesting as GDM. This hypothesis is consistent with previous studies in which type 2 diabetes has been associated with abnormalities in SQ AT levels of the IGF-1 receptor and IGFBP3 [50]. While our current study is limited by sample size and by differences in age and BMI between groups, the large magnitude of the results presented support a new model to explore the mechanism of AT expansion in pregnancy and its role in the aetiology of GDM.

## Electronic supplementary material

ESM Table 1 (PDF 468 kb)

ESM Fig. 1 (PDF 5535 kb)

ESM Fig. 2 (PDF 5484 kb)

ESM Fig. 3 (PDF 7815 kb)

## Abbreviations

AT: Adipose tissue
GDM: Gestational diabetes mellitus
IGFBP: IGF binding protein
NGT: Normal glucose tolerance
OM: Omental
PAPP-A: Pregnancy-associated plasma protein-A
SQ: Subcutaneous

## References

[1] Sacks DA, Hadden DR, Maresh M (2012). Frequency of gestational diabetes mellitus at collaborating centers based on IADPSG consensus panel-recommended criteria: the Hyperglycemia and Adverse Pregnancy Outcome (HAPO) Study. Diabetes Care.

[2] Getahun D, Nath C, Ananth CV, Chavez MR, Smulian JC (2008). Gestational diabetes in the United States: temporal trends 1989 through 2004. Am J Obstet Gynecol.

[3] Albrecht SS, Kuklina EV, Bansil P (2010). Diabetes trends among delivery hospitalizations in the U.S., 1994–2004. Diabetes Care.

[4] Moore Simas TA, Doyle Curiale DK, Hardy J, Jackson S, Zhang Y, Liao X (2010). Efforts needed to provide Institute of Medicine-recommended guidelines for gestational weight gain. Obstet Gynecol.

[5] Metzger BE (2007). Long-term outcomes in mothers diagnosed with gestational diabetes mellitus and their offspring. Clin Obstet Gynecol.

[6] Hillier TA, Pedula KL, Schmidt MM, Mullen JA, Charles MA, Pettitt DJ (2007). Childhood obesity and metabolic imprinting: the ongoing effects of maternal hyperglycemia. Diabetes Care.

[7] Malcolm J (2012). Through the looking glass: gestational diabetes as a predictor of maternal and offspring long-term health. Diabetes Metab Res Rev.

[8] Kjos SL, Peters RK, Xiang A, Henry OA, Montoro M, Buchanan TA (1995). Predicting future diabetes in Latino women with gestational diabetes. Utility of early postpartum glucose tolerance testing. Diabetes.

[9] Cheung NW, Byth K (2003). Population health significance of gestational diabetes. Diabetes Care.

[10] Gray SL, Vidal-Puig AJ (2007). Adipose tissue expandability in the maintenance of metabolic homeostasis. Nutr Rev.

[11] Virtue S, Vidal-Puig A (2010). Adipose tissue expandability, lipotoxicity and the metabolic syndrome – an allostatic perspective. Biochim Biophys Acta.

[12] Gupta D, Krueger CB, Lastra G (2012). Over-nutrition, obesity and insulin resistance in the development of beta-cell dysfunction. Curr Diabetes Rev.

[13] Summers SA (2006). Ceramides in insulin resistance and lipotoxicity. Prog Lipid Res.

[14] Kim JY, van de Wall E, Laplante M (2007). Obesity-associated improvements in metabolic profile through expansion of adipose tissue. J Clin Invest.

[15] McLaughlin T, Lamendola C, Liu A, Abbasi F (2011). Preferential fat deposition in subcutaneous versus visceral depots is associated with insulin sensitivity. J Clin Endocrinol Metab.

[16] Tchernof A, Despres JP (2013). Pathophysiology of human visceral obesity: an update. Physiol Rev.

[17] Alligier M, Gabert L, Meugnier E (2013). Visceral fat accumulation during lipid overfeeding is related to subcutaneous adipose tissue characteristics in healthy men. J Clin Endocrinol Metab.

[18] Herrera E (2000). Metabolic adaptations in pregnancy and their implications for the availability of substrates to the fetus. Eur J Clin Nutr.

[19] Ryan EA, O’Sullivan MJ, Skyler JS (1985). Insulin action during pregnancy. Studies with the euglycemic clamp technique. Diabetes.

[20] Catalano PM (2014). Trying to understand gestational diabetes. Diabet Med.

[21] Kirwan JP, Hauguel-De Mouzon S, Lepercq J (2002). TNF-alpha is a predictor of insulin resistance in human pregnancy. Diabetes.

[22] Laursen LS, Overgaard MT, Soe R (2001). Pregnancy-associated plasma protein-A (PAPP-A) cleaves insulin-like growth factor binding protein (IGFBP)-5 independent of IGF: implications for the mechanism of IGFBP-4 proteolysis by PAPP-A. FEBS Lett.

[23] Beattie J, Allan GJ, Lochrie JD, Flint DJ (2006). Insulin-like growth factor-binding protein-5 (IGFBP-5): a critical member of the IGF axis. Biochem J.

[24] Garten A, Schuster S, Kiess W (2012). The insulin-like growth factors in adipogenesis and obesity. Endocrinol Metab Clin N Am.

[25] Huang L, Liu J, Feng L, Chen Y, Zhang J, Wang W (2014). Maternal prepregnancy obesity is associated with higher risk of placental pathological lesions. Placenta.

[26] Jones CJ, Fox H (1976). Placental changes in gestational diabetes. An ultrastructural study. Obstet Gynecol.

[27] Mele J, Muralimanoharan S, Maloyan A, Myatt L (2014). Impaired mitochondrial function in human placenta with increased maternal adiposity. Am J Physiol Endocrinol Metab.

[28] O’Tierney-Ginn P, Presley L, Myers S, Catalano P (2015). Placental growth response to maternal insulin in early pregnancy. J Clin Endocrinol Metab.

[29] Landy HJ, Gomez-Marin O, O’Sullivan MJ (1996). Diagnosing gestational diabetes mellitus: use of a glucose screen without administering the glucose tolerance test. Obstet Gynecol.

[30] Atilano LC, Lee-Parritz A, Lieberman E, Cohen AP, Barbieri RL (1999). Alternative methods of diagnosing gestational diabetes mellitus. Am J Obstet Gynecol.

[31] Gealekman O, Guseva N, Hartigan C (2011). Depot-specific differences and insufficient subcutaneous adipose tissue angiogenesis in human obesity. Circulation.

[32] Schindelin J, Arganda-Carreras I, Frise E (2012). Fiji: an open-source platform for biological-image analysis. Nat Methods.

[33] Rojas-Rodriguez R, Gealekman O, Kruse ME (2014). Adipose tissue angiogenesis assay. Methods Enzymol.

[34] Hoffstedt J, Arner E, Wahrenberg H (2010). Regional impact of adipose tissue morphology on the metabolic profile in morbid obesity. Diabetologia.

[35] Veilleux A, Caron-Jobin M, Noel S, Laberge PY, Tchernof A (2011). Visceral adipocyte hypertrophy is associated with dyslipidemia independent of body composition and fat distribution in women. Diabetes.

[36] Bouloumie A, Lolmede K, Sengenes C, Galitzky J, Lafontan M (2002). Angiogenesis in adipose tissue. Ann Endocrinol (Paris).

[37] Hausman GJ, Richardson RL (2004). Adipose tissue angiogenesis. J Anim Sci.

[38] Tran KV, Gealekman O, Frontini A (2012). The vascular endothelium of the adipose tissue gives rise to both white and brown fat cells. Cell Metab.

[39] Divoux A, Tordjman J, Lacasa D (2010). Fibrosis in human adipose tissue: composition, distribution, and link with lipid metabolism and fat mass loss. Diabetes.

[40] Henegar C, Tordjman J, Achard V (2008). Adipose tissue transcriptomic signature highlights the pathological relevance of extracellular matrix in human obesity. Genome Biol.

[41] Keller P, Gburcik V, Petrovic N (2011). Gene-chip studies of adipogenesis-regulated microRNAs in mouse primary adipocytes and human obesity. BMC Endocr Disord.

[42] Tchoukalova YD, Votruba SB, Tchkonia T, Giorgadze N, Kirkland JL, Jensen MD (2010). Regional differences in cellular mechanisms of adipose tissue gain with overfeeding. Proc Natl Acad Sci U S A.

[43] Kim JI, Huh JY, Sohn JH (2015). Lipid-overloaded enlarged adipocytes provoke insulin resistance independent of inflammation. Mol Cell Biol.

[44] Lu Q, Li M, Zou Y, Cao T (2014). Induction of adipocyte hyperplasia in subcutaneous fat depot alleviated type 2 diabetes symptoms in obese mice. Obesity (Silver Spring).

[45] Han J, Lee JE, Jin J (2011). The spatiotemporal development of adipose tissue. Development.

[46] Tang W, Zeve D, Suh JM (2008). White fat progenitor cells reside in the adipose vasculature. Science.

[47] Spencer M, Unal R, Zhu B (2011). Adipose tissue extracellular matrix and vascular abnormalities in obesity and insulin resistance. J Clin Endocrinol Metab.

[48] Xin X, Rodrigues M, Umapathi M (2013). Hypoxic retinal Muller cells promote vascular permeability by HIF-1-dependent up-regulation of angiopoietin-like 4. Proc Natl Acad Sci U S A.

[49] Gealekman O, Gurav K, Chouinard M (2014). Control of adipose tissue expandability in response to high fat diet by the insulin-like growth factor-binding protein-4. J Biol Chem.

[50] Yamada PM, Mehta HH, Hwang D, Roos KP, Hevener AL, Lee KW (2010). Evidence of a role for insulin-like growth factor binding protein (IGFBP)-3 in metabolic regulation. Endocrinology.

