# Supplementary material for: Human adipose tissue expansion in pregnancy is impaired in gestational diabetes mellitus
Source: Diabetologia. 2015 Jun 13;58(9):2106–14. doi: 10.1007/s00125-015-3662-0 (PMC4526585; doi:10.1007/s00125-015-3662-0)
Supplement: Supplementary file 1 — (PDF 468 kb) [file 125_2015_3662_MOESM1_ESM.pdf]

KEGG Enrichment			
Probes with KEGG annotations in above list: 188			
The chip holds 5870 probes annotated to 229 pathways			
**Gene List**	**Path**	**Path Name**	**P-val**
⇓ DNAJA2 OS9 CKAP4 CRYAB ERN1 SEC61G UBQLN1 STT3A M AP3K5PRKCSH SKP1 SSR1 SSR3 UBE2D1 XBP1 CAPN1 MBTPS1 PDIA4	4141	Protein processing in endoplasmic reticulum	4.77E-08
⇓ MYL9 COL6A1 COL6A2 CTNNB1 FYN PARVB ILK ITGA7 ITGB5 JUNPDGFA PPP1CB MAPK3 SHC1 THBS1 TLN1 ZYX	4510	Focal adhesion	4.35E-06
⇓ FBXO5 PPP1CB PPP2CA PPP2R1A PPP3R1 MAPK3 SKP1 YWH AECAMK2G SMC3 CDC27	4114	Oocyte meiosis	3.39E-05
⇓ CTNNB1 JUN CYCS MAPK3 TCF7L2 TGFB1 TGFB2	5210	Colorectal cancer	0.00019882
⇓ SEC61G SRP68 SRPR SEC11C	3060	Protein export	0.00027554
⇓ GNAS JUN PPP2CA PPP2R1A MAPK3 MAPK13 TGFB1 TGFB2 CFLAR	5142	Chagas disease (American trypanosomiasis)	0.00038197
⇓ CTBP1 CTNNB1 DAAM1 JUN LRP5 PPP2CA PPP2R1A PPP3R1 SKP1TCF7L2 CAMK2G	4310	Wnt signaling pathway	0.00055785
⇓ CTNNB1 FYN MAPK3 PTPN1 PVRL2 TCF7L2 IQGAP1	4520	Adherens junction	0.0006184
⇓ NEDD4L SKP1 UBA1 UBE2D1 UBE2L3 UBE2N ITCH CUL4A CU L7CDC27	4120	Ubiquitin mediated proteolysis	0.00081475
⇓ MYL9 CFL1 GSN ITGA7 ITGB5 PDGFA PPP1CB ENAH MAPK3P IP4K2B IQGAP1 ARHGEF7 ARHGEF1	4810	Regulation of actin cytoskeleton	0.00139422
⇓ PPP2CA PPP2R1A MAPK3 SKP1 TGFB1 TGFB2 THBS1	4350	TGF-beta signaling pathway	0.00157004
⇓ JUN ARHGDIB MAP3K5 NTRK2 MAPK3 MAPK13 SHC1 YWHA ECAMK2G	4722	Neurotrophin signaling pathway	0.00182166

Supplementary Table 1: Kyoto Encyclopaedia of Genes and Genomes (KEGG) pathways enriched in genes differentially expressed in OM and SQ adipose tissue from non-pregnant compared to pregnant subjects. Genes that were significantly different with a P value of < 0.0001 were used for the analysis.
