# Supplementary material for: Human adipose tissue expansion in pregnancy is impaired in gestational diabetes mellitus
Source: Diabetologia. 2015 Jun 13;58(9):2106–14. doi: 10.1007/s00125-015-3662-0 (PMC4526585; doi:10.1007/s00125-015-3662-0)
Supplement: Supplementary file 2 — (PDF 5535 kb) [file 125_2015_3662_MOESM2_ESM.pdf]

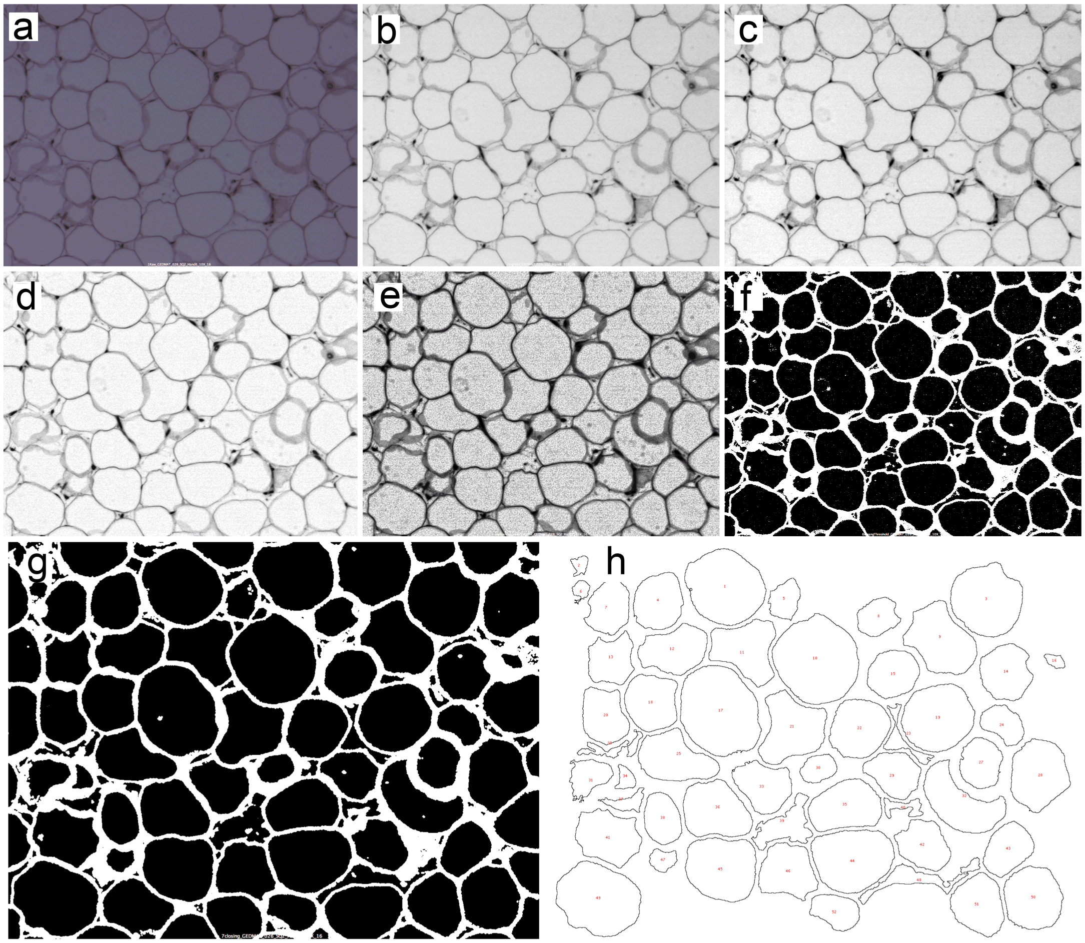

Supplementary Figure 1: Image processing and quantification of human adipose tissue (AT) sections. AT sample was fixed in 4% paraformaldehyde overnight and rinsed in phosphate buffered solution (PBS). Sample was embedded in paraffin, cut in 8μm sections, and mounted on Superfrost Plus microscope slides (Fisher Scientific). **a.** AT section stained with hematoxylin and eosin (H&E). 10X images were taken using brightfield microscopy (Zeiss Axiovert 35) and AxioCam Icc 1 digital camera (Zeiss). **b.** Image is imported to ImageJ software and converted to 8-bit grayscale image. **c.** Enhancement of image contrast (Process/Enhance Contrast/ Saturated pixels: 0.35%;normalize). **d.** Background subtraction (Process/subtract background/rolling ball radius=50.0 pixels;Light background;sliding paraboloid). **e.** Enhancement of image contrast (Process/Enhance Contrast/ Saturated pixels: 10 %; normalize). **f.** Binarized image (Image/adjust/threshold/Huang;dark background;apply). **g.** Processing on the binary image fills in small holes (Process/Binary/fill holes) **h.** Resulting image showing the outlines of the counted objects ("Analyze Particles...", "size=1200-Infinity circularity=0.10-1.00 show=Outlines; display results; exclude on edges"). Note the reference numbers for each counted adipocyte, which is associated with corresponding area in results sheet.
