# Supplementary material for: Human adipose tissue expansion in pregnancy is impaired in gestational diabetes mellitus
Source: Diabetologia. 2015 Jun 13;58(9):2106–14. doi: 10.1007/s00125-015-3662-0 (PMC4526585; doi:10.1007/s00125-015-3662-0)
Supplement: Supplementary file 3 — (PDF 5484 kb) [file 125_2015_3662_MOESM3_ESM.pdf]

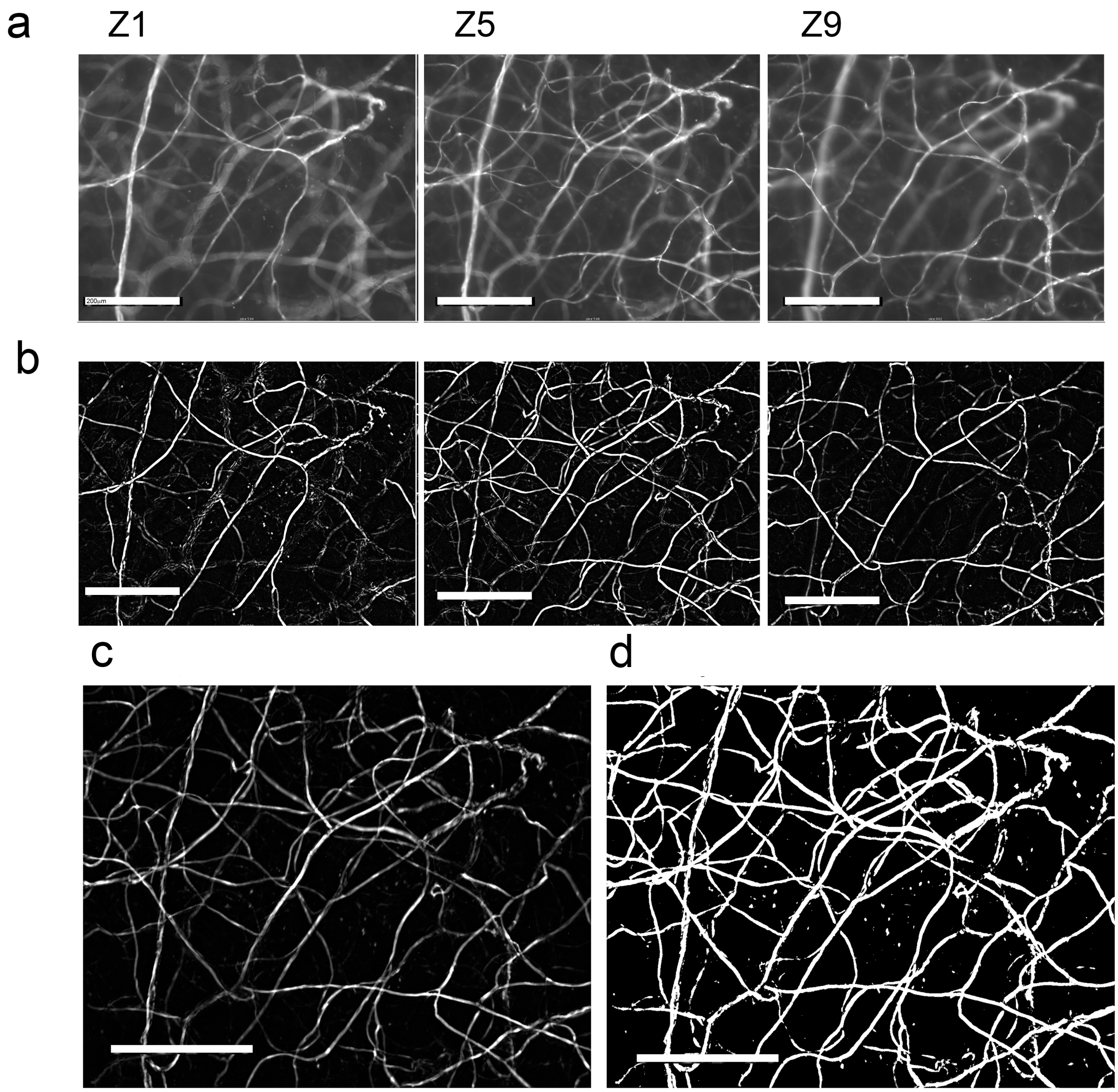

Supplementary Figure 2: Image processing and quantification of capillary density from human wholemount adipose tissue (AT). AT sample was cut into small pieces (250mm³) and stained with rhodamine labelled lectin (UEA1, Vector). **a.** Selected Z-planes illustrating raw images acquired with a Zeiss AxioCam HR (1300x1030 pix).. **b.** Image stack is imported into ImageJ (FIJI) software and the background is subtracted (Process/subtract background/rolling ball radius=5.0 pixels). **c.** A maximal intensity projection of the Z-stack is generated (Image/Stacks/Z Project/Max intensity). **d.** Greyscale image is converted into a binary image (Process/Binary/Make Binary Method:Default, Background: Dark). The sum of white pixels in the image are recorded (plugins/Feature Extraction/Feature J/ Feature J statistics/mass), and expressed as the percent of the total pixels in the image (mass/255)(100)/(1339000).
