# Supplementary material for: Human adipose tissue expansion in pregnancy is impaired in gestational diabetes mellitus
Source: Diabetologia. 2015 Jun 13;58(9):2106–14. doi: 10.1007/s00125-015-3662-0 (PMC4526585; doi:10.1007/s00125-015-3662-0)
Supplement: Supplementary file 4 — (PDF 7815 kb) [file 125_2015_3662_MOESM4_ESM.pdf]

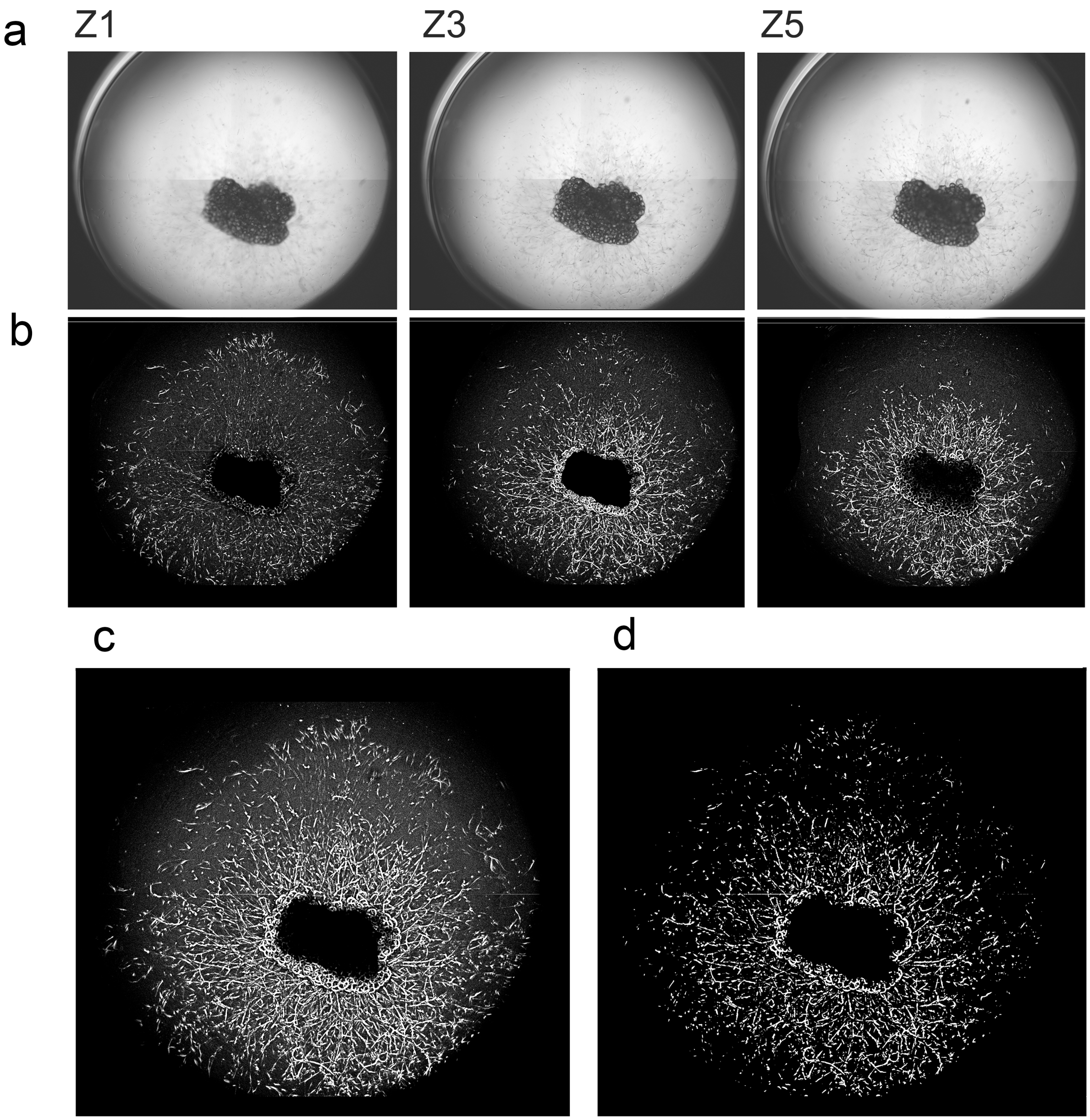

Supplementary Figure 3: Image processing and quantification of angiogenic potential from human adipose tissue explant. AT biopsy was cut into small (1mm³) pieces, after obvious vasculature and fibrous tissue were removed. Individual explants (AT pieces) were embedded in individual wells of a 96-multiwell plate. After 11 days of culture, Micromanager software was used to operate the automated stage of the Zeiss Axio Observer Z1 equipped with a Clara High Resolution CCD Camera (Andor) and record the positions of each well. Images were taken as a canvas of four quadrants per well, and then combined into a single image. **a.** AT explant imaged under 2.5 magnification as a stack composed of 5 z planes at 150μm intervals. **b. Same Z-planes after** Background is subtracted from the stack (Process/subtract background/rolling ball radius=5.0 pixels). **c.** Maximal intensity projection of the Z-stack (Image/Stacks/Z Project/Max intensity). **d.** Binarized image (Process/Binary/Make Binary Method:Default, Background: Dark). The resulting number of pixels are recorded for each image (plugins/Feature Extraction/Feature J/ Feature J statistics).
